# The *GhTT2_A07* gene is linked to the brown colour and natural flame retardancy phenotypes of *Lc1* cotton (*Gossypium hirsutum* L.) fibres

**DOI:** 10.1093/jxb/erw312

**Published:** 2016-08-27

**Authors:** Doug J. Hinchliffe, Brian D. Condon, Gregory Thyssen, Marina Naoumkina, Crista A. Madison, Michael Reynolds, Christopher D. Delhom, David D. Fang, Ping Li, Jack McCarty

**Affiliations:** ^1^Cotton Chemistry and Utilization Research Unit, Southern Regional Research Center, Agricultural Research Service, USDA, 1100 Robert E. Lee Blvd, New Orleans, LA 70124, USA; ^2^Cotton Fiber Bioscience Research Unit, Southern Regional Research Center, Agricultural Research Service, USDA, 1100 Robert E. Lee Blvd, New Orleans, LA 70124, USA; ^3^Cotton Structure and Quality Research Unit, Southern Regional Research Center, Agricultural Research Service, USDA, 1100 Robert E. Lee Blvd, New Orleans, LA 70124, USA; ^4^Genetics and Sustainable Agriculture Research Unit, Agricultural Research Service, USDA, Mississippi State, MS 39762, USA

**Keywords:** Lc1 locus, brown fibre, cotton, fame retardant, flavonoid, proanthocyanidin, textiles, transparent testa.

## Abstract

Some naturally coloured brown cotton fibres from accessions of *Gossypium hirsutum* L. can be used to make textiles with enhanced flame retardancy (FR). Several independent brown fibre loci have been identified and mapped to chromosomes, but the underlying genes have not yet been identified, and the mechanism of lint fibre FR is not yet fully understood. In this study, we show that both the brown colour and enhanced FR of the *Lc1* lint colour locus are linked to a 1.4Mb inversion on chromosome A07 that is immediately upstream of a gene with similarity to Arabidopsis TRANSPARENT TESTA 2 (TT2). As a result of the alternative upstream sequence, the transcription factor *GhTT2_A07* is highly up-regulated in developing fibres. In turn, genes in the phenylpropanoid metabolic pathway are activated, leading to biosynthesis of proanthocyanidins and accumulation of inorganic elements. We show that enhanced FR and anthocyanin precursors appear in developing brown fibres well before the brown colour is detectible, demonstrating for the first time that the polymerized proanthocyanidins that constitute the brown colour are not the source of enhanced FR. Identifying the particular colourless metabolite that provides *Lc1* cotton with enhanced FR could help minimize the use of synthetic chemical flame retardant additives in textiles.

## Highlight

The brown fibre cotton *Lc1* locus is linked to a 1.4Mb genomic inversion that activates *GhTT2_A07*. This mutation upregulates flavonoid biosynthesis and confers natural flame retardancy.

## Introduction

Naturally coloured cotton fibres exist in various hues including light to dark brown, red, rust, and green, and are found among new world tetraploid *Gossypium* species including *G. hirsutum* L., *G. barbadense* L., *G. darwinii* G. Watt, and *G. mustelinum* Miers ex G. Watt., as well as their diploid progenitors *G. arboreum* L. and *G. herbaceum* L. Studies on the inheritance of brown lint colour in Upland cottons date back to the early 20th century when it was determined that plants producing fibre in various shades of brown and green were allelomorphic to plants producing white fibres, with F_1_ plants producing an intermediate fibre colour phenotype (Ware, 1932). In a cross with a brown fibre phenotype and white fibre phenotype the fibre colour segregation in the F_2_ generation was 1:2:1 as brown, intermediate brown, and white (Ware, 1932). Currently, researchers have characterized at least six genetic loci that are linked to brown fibre cotton (*Lc1*–*Lc6*) and result in various shades from light to dark brown. These genetic loci were demonstrated to be incompletely dominant to white cotton (Endrizzi and Kohel, 1966; Kohel, 1985). More recently, The *Lc1* locus of *G. hirsutum* was mapped to within 3.8 cM on the long arm of chromosome A07, and the *Lc2* locus was mapped to within 4.4 cM on the short arm of chromosome A06 using simple sequence repeat (SSR) and expressed sequence tag (EST)-SSR markers (Wang *et al.*, 2014).

Naturally coloured cottons are typically grown as a source of fibre for niche textile markets that promote the use of natural colours in textiles as an alternative to scouring, bleaching, and dyeing cotton fibres. This use has great merit considering that global textile processing generates a large toxic chemical waste stream with negative environmental impacts (Khatri *et al.*, 2015). Despite the appeal of naturally coloured cottons as an environmentally friendly source of staple fibre for the textile industry, they still occupy only a small market due to inferior agronomic traits. The fibres of brown cotton in particular are typically lower yielding, shorter, and weaker compared with conventional white fibres, which causes difficulty during modern high speed textile processing into yarns, threads, and woven fabrics due to breakage and fibre loss (Dutt *et al.*, 2004; Yuan *et al.*, 2013).

Another limitation to the commercialization of naturally coloured cottons is the lack of colour uniformity and diversity. To that end, and to address negative correlations with fibre quality traits, a large number of studies have been conducted to elucidate the biosynthetic pathways leading to colour formation in brown fibre cottons and determine the source of brown colour. The overall results have demonstrated upregulation of structural genes in the phenylpropanoid and flavonoid biosynthetic pathways and proanthocyanidins (PAs), or condensed tannins, as the source of the brown colour (Xiao *et al.*, 2007; Li *et al.*, 2012; Feng *et al.*, 2013; Gong *et al.*, 2014; Xiao *et al.*, 2014). This was initially demonstrated through dimethylaminocinnamaldehyde (DMACA) staining of brown fibres compared with white fibres from different cotton lines (Xiao *et al.*, 2007). It was also demonstrated through DMACA and toluidine blue O (TBO) staining that PA precursors begin to accumulate as early as 3 days post-anthesis (DPA) in developing brown fibres (Li *et al.*, 2012). Concurrent with staining techniques the presence of flavonoids during brown fibre developmental time points, structural genes in the early and late committed PA biosynthetic pathways were shown by semi-quantitative PCR to be upregulated in developing brown fibres compared with white fibres with peak expression levels during the elongation stage of fibre development (Xiao *et al.*, 2007; Li *et al.*, 2012). Upregulation of the flavanoid pathway in brown cotton fibres was further confirmed by detection of elevated levels of specific flavanoids by high performance liquid chromotagraphy (HPLC) in developing brown cotton fibres (Feng *et al.*, 2013). Further studies have utilized transcriptome analysis by RNA-seq to demonstrate flavonoid structural gene upregulation in developing brown cotton fibres (Gong *et al.*, 2014; Xiao *et al.*, 2014). It was postulated based on liquid chromatography–mass spectrometry (LC-MS) analysis of PA extracts from 20 DPA developing brown and white fibres that leucoanthocyanidin reductase (LAR) represents the primary flow of the PA pathway in brown fibres with catechin and gallocatechin providing the primary precursors to condensed tannins (Xiao *et al.*, 2014). Conversely, it was indicated by matrix-assisted laser desorption/ionization time of flight mass spectrometry (MALDI-TOF MS) analysis of brown and white cotton fibres that anthocyanidins reductase (ANR) was a key structural gene in the brown fibre PA pathway based on detection of epigallocatechin and epicatechin precursors in 20 DPA developing brown fibres (Feng *et al.*, 2014). Taken together, most of these results have demonstrated the source of the pigmentation in brown cotton fibre to be PAs in fibres of the brown cotton lines. However, it has also been proposed that quinones are the source of colour development in brown cotton fibres and accumulate due to oxidation of PAs (Feng *et al.*, 2014). Regardless of the source of colour in brown fibres, the unifying finding was upregulation of structural genes in the metabolic pathways leading to PA biosynthesis. Interestingly, no analyses have been conducted on the regulatory genes that are known to control structural gene expression in the committed PA pathway and which have been extensively studied in Arabidopsis and well reviewed in Xu *et al.* (2014*a*).

The interest of our laboratory in brown cotton fibres is presently focused on the enhanced flame retardancy (FR) of brown cotton fibres and fabrics (Fox, 1996; VanZandt *et al.*, 1997; Kimmel *et al.*, 2000; Parmar and Chakraborty, 2001; Corradini *et al.*, 2009; Zhang *et al.*, 2009; Hinchliffe *et al.*, 2015; Nam *et al.*, 2016). In its current state, this level of FR has specific end-use applications including automobile interiors where the burn rates of materials are limited according to standardized testing procedures (ASTM D5132-11; ASTM, 2011). Woven and needlepunched nonwoven fabrics produced from commercially available brown fibres were recently demonstrated to pass standardized testing for automotive interiors (Fox, 1996; VanZandt *et al.*, 1997; Hinchliffe *et al.*, 2015). The brown cotton FR also has the potential to reduce the amount of FR chemical additives required for textiles to pass more stringent tests such as those required by the Federal Aviation Administration for aircraft interior components (FAA, 1986).

The source of brown fibre FR is not yet understood and is not absolutely correlated to the intensity of colour in different cultivars (Hinchliffe *et al.*, 2015). Recently, our laboratory demonstrated that the greater FR observed in brown naturally coloured cottons is likely the result of inorganic salts such as sodium being sequestered through ionic interactions with the partial negative charges generated by adjacent hydroxyl units on the B-ring of flavonoid units in PAs and the possible formation of flavonoid–metal complexes (Nam *et al.*, 2016). The previous studies conducted by Hinchliffe *et al.* (2015) and Nam *et al.* (2016) indicate a synergistic process of enhanced FR in brown cotton fibres. Based on these observations, elucidating the regulatory mechanisms of the cotton brown fibre PA and upstream precursor biosynthetic pathways, and identification of the causative brown fibre mutation will provide greater insight to the molecular basis of enhanced FR in brown fibres.

Here we report the results of (i) characterization of the combustion properties of developing and mature fibres and nonwoven fabrics derived from two white fibre and two *Lc1* brown fibre cotton lines; (ii) fibre transcriptome analyses of developing white and brown cotton fibres by mRNA-seq and RT-qPCR, which revealed upregulation of many of the structural genes and regulatory genes in the PA and upstream precursor biosynthetic pathways; (iii) identification of the causative mutation by next-generation genomic sequencing and genetic mapping as a 1.4Mb sequence inversion directly upstream of the *GhTT2_A07* flavonoid transcription factor gene.

## Materials and methods

### Cotton lines and cultivation

A brown fibre cotton (*G. hirsutum*) line of unknown background was observed to have a reversion in fibre colour from brown to white on one branch. White and brown fibre seed cotton was collected from this single plant and homozygous lines, respectively designated MC-WL and MC-BL, were advanced and maintained in Starkville, MS. Both lines continued to breed true for their respective fibre colours. The lines were further advanced by forced self-pollination single seed descent (SSD) for three generations in New Orleans, LA. The cotton lines PD-3 (Culp *et al.*, 1988) and PD 93002 (May *et al.*, 1994) were planted and advanced by SSD. The cotton line PD-3 is a conventional cotton variety with white fibres. The cotton line PD 93002 is an improved fibre quality brown cotton derived from a cross of an unknown brown fibre cotton with PD-3 followed by single plant selections in the F_2_ generation (May *et al.*, 1994). Pure lines of MC-WL, MC-BL, PD-3, and PD 93002 were planted in an experimental plot in New Orleans, LA in 2012 and 2013 and grown using standard agricultural practices for cotton cultivation. The soil type in the field was Aquents dredged over alluvian in an elevated location to provide for proper drainage. The parent lines MC-BL and MC-WL were crossed, and the parent lines PD-3 and PD 93002 were crossed to produce two F_2_ populations of 496 and 99 individuals, respectively, that were planted in the same experimental plot in New Orleans, LA in 2014 and 2015 along with the parent lines. Plants that were grown under greenhouse conditions were cultivated in 18.9L pots with Metro-Mix 366 potting soil (Sun Gro Horticulture Canada Ltd, Agawam, MA, USA).

### Nonwoven cotton fabric production

Cotton fibres were collected from plants grown in the 2012 growing season and the fibres were converted into hydroentangled nonwoven fabrics on a 1 m wide Fleissner pilot-scale hydroentanglement system (Trützschler Nonwovens GmbH, Dülmen, Germany) running at a constant production speed of 5 m min^−1^. The hydroentanglement system utilized three pressure heads: one low pressure for fabric wet-out maintained at a constant pressure of 3MPa during fabric production; and two high-pressure heads both maintained at 10MPa during fabric production. Each strip on the pressure heads consisted of 16 orifices per centimetre with an orifice pore size of 120µm. The water used for the hydroentangled fabric production was ambient temperature, which was approximately 25 °C. After hydroentanglement, the fabrics were fed directly through a gas-fired fabric drying oven (Trützschler Nonwovens GmbH) at ~170 °C and wound into rolls.

### Flammability testing

The flammability characteristics of the hydroentangled fabrics manufactured from brown and white cotton fibres were determined using a 45 ° flammability tester (Model TC 45, Govmark Ltd) according to test method ASTM D1230-10 (ASTM, 2010) with the following modifications to the protocol. The exposure time of the flame source to the test specimen was increased from the 1s exposure time specified in the test method to 10s. This was necessary to achieve ignition of the white greige fibre hydroentangled fabrics and establish a baseline flame exposure time. Five replications were performed for fabrics manufactured from each cotton line.

### Microscale combustion calorimetry and fibre DMACA staining

Fibres from the four cotton lines were tested for enhanced flame retardancy using microscale combustion calorimetry (MCC), which is known to correlate with flammability of textiles and is used as a standardized test by the Federal Aviation Administration for aircraft interior components (ASTM D7309-13; FAA, 1986; Lyon *et al.*, 2007; ASTM, 2013). Approximately 4mg of cotton fibre was placed in an MCC ceramic specimen cup, and the sample mass measured prior to and after pyrolysis on a Sartorius CP2P-F micro balance (Satorius, Bohemia, NY, USA) stationed on a Scienceware Vibrasorb vibration damping mount (Bel-Art Products, Wayne, NJ, USA) mounted on a marble slab table. The heat release capacity (HRC: Jg^−1^ K^−1^), peak heat release rate (pHRR: W g^−1^), total heat release (tHR: kJg^−1^), temperature at pHRR (°C), and percentage mass of sample not consumed (% char yield) were measured on an MCC model MCC-2 (Deatak, McHenry, IL, USA) and analysed using the MCC Curve Fit v.2 software (Deatak). The specimen heating rate was held constant at 1.2 °C s^−1^ and data were recorded from 90 to 550 °C. For analysis of immature fibres, developing bolls were harvested in the greenhouse at 20, 28, 36, and 44 DPA and the carpels removed leaving the locules in place. To detect the presence of flavonoids, 50 µl of DMACA (Becton, Dickinson and Co., Sparks, MD, USA) was carefully distributed onto the surface of one to two locules and incubated for 5min at room temperature before image acquisition. Unstained locules were removed from the boll and fibres hand ginned from the ovules. The fibres were dried at room temperature and used for MCC analyses as described.

### Colourspace measurement of cotton fibres

The coloration of naturally coloured cotton and white cotton fibres were measured in CIE (International Commission on Illumination, Vienna, Austria) L*a*b* colour space (Ibraheem, 2012), which includes all colours perceivable to the human eye. In CIE L*a*b* colour space, L* indicates whiteness (0=black; 100=white); a* indicates colours from greenish (negative values) to reddish (positive values); and b* indicates colours from bluish (negative values) to yellowish (positive values).

### Sample collection, RNA, and DNA isolations

Mature seed cotton in all years from the parent cotton lines and F_2_ population was hand-harvested and ginned using a laboratory roller gin. During the 2012 growing season, fibre samples representing developmental time points were collected from the four cotton lines at 6, 8, 10, 12, 14, 16, 18, and 20 DPA as previously described (Hinchliffe *et al.*, 2010). Total RNA was extracted and an on-column DNase I digestion performed using the Sigma Spectrum Plant Total RNA Kit (Sigma-Aldrich, St Louis, MO, USA) as per the manufacturer’s protocol and RNA quantity and integrity evaluated as previously described (Hinchliffe *et al.*, 2011). Genomic DNA from the cotton parents and mapping populations was isolated from young leaf tissues as previously described (Fang *et al.*, 2010).

### DNA sequencing and linkage mapping

Genomic DNA from the parents used in this study, MC-WL, MC-BL, PD-3, and PD-93002, was isolated from ~100mg of fresh root radical tissue as previously described (Fang *et al.*, 2010) and used for Illumina HiSeq X Ten Sequencing (Novogene Corp., Chula Vista, CA, USA) using 150bp paired-end sequencing runs. Equal amounts of the four indexed libraries were run together on three lanes of an Illumina flow cell. Sequence reads were aligned to the draft *G. hirsutum* TM-1 reference genome as well as a pseudo-tetraploid genome composed of the reference sequences from two diploids, *Gossypium raimondii* Ulbr., and *G. arboreum* (Paterson *et al.*, 2012; Li *et al.*, 2014; Zhang *et al.*, 2015) with GSNAP software (Wu and Nacu, 2010). Polymorphisms were identified with bcftools software and by manual inspection of alignment files (Li *et al.*, 2009; Li, 2011). Select single nucleotide polymorphisms (SNPs) were converted to subgenome specific primers and scored on the F_2_ populations as before (Thyssen *et al.*, 2014), which also allowed detection of the structural variation using primers flanking the boundaries of the 1.4Mb inversion (Supplementary Tables S1 and S2 at *JXB* online).

### Gene expression analysis

Total RNA from 8 and 20 DPA MC-BL and MC-WL cotton fibres harvested in 2012 were utilized for library preparation and high-throughput sequencing by mRNA-seq (LC Sciences, Houston, TX, USA). Two biological replications for each cotton line and developmental time point were used for a total of eight samples. Sample preparation and library constructions was performed using TruSeq Stranded mRNA Library Prep Kit (Illumina Inc., San Diego, CA, USA) as per the manufacturer’s protocols. Samples were sequenced using a HiSeq 2000 (Illumina Inc.) with 100bp paired-end reads. Equal amounts of the eight indexed libraries were run together on two lanes of an Illumina flow cell. Raw sequence reads were filtered for quality and trimmed by SICKLE (Joshi and Fass, 2011) and aligned to the draft *G. hirsutum* TM-1 reference genome (Zhang *et al.*, 2015) with GSNAP software (Wu and Nacu, 2010). Reads mapping to annotated genes were counted using BEDTools software (Quinlan and Hall, 2010). Differential expression was determined by data normalization and ANOVA as previously described (Naoumkina *et al.*, 2014). Selected genes from the flavonoid pathway were studied with RT-qPCR using specific primers (Supplementary Table S3) according to standard procedures (Bustin *et al.*, 2009). The reference genes used in the RT-qPCR reactions were the 18S rRNA (GenBank accession U42827), ubiquitin-conjugating protein E2 (GhUCP E2; GenBank AI730710), and α-tubulin 4 (GhTubA4; GenBank AF106570) (Whittaker and Triplett, 1999).

### Elemental analysis using inductively coupled plasma mass spectrometry (ICP-MS)

Mature fibre samples from the cotton lines MC-BL, MC-WL, PD 93002, and PD-3 were collected from the field during the 2012 and 2013 growing seasons. Triplicate samples for each line from each year were digested in 8M TraceMetal Grade nitric acid (Thermo Fisher Scientific Inc., Waltham, MA, USA) and analysed by ICP-MS as previously described (Hinchliffe *et al.*, 2015). The ICP-MS analysis was performed by the Nanomedicines Characterization Core Facility (University of North Carolina, Chapel Hill, NC, USA) using a Nexion 300-D ICP-MS (Perkin Elmer, Waltham, MA, USA).

## Results

### Inherent flame retardancy of naturally coloured brown cottons

The MCC analysis revealed significantly lower heat release capacity, peak heat release rate, and total heat release from fibres of both brown cotton lines compared with the white lines and indicated enhanced FR properties of brown cotton fibres that were consistent with previous studies by this laboratory using commercially available brown and white cotton fibres and nonwoven fabrics (Hinchliffe *et al.*, 2015; Table 1). The percentage char yield, which is a measurement of inorganic compounds remaining after pyrolysis, was also significantly higher from fibres of both brown lines (MC-BL and PD 93002) compared with the white lines (MC-WL and PD 93002). This was previously demonstrated to correlate with higher levels of inorganic salts, which accumulated synergistically with condensed tannins and resulted in greater FR in cotton brown fibres compared with white fibres (Nam *et al.*, 2016). The fibre colour phenotypes were distinguished in CIE L*a*b* colour space (Table 1). These data confirmed our selection of the cotton lines for further studies on the genetics of fibre colour and linkage with enhanced FR in brown fibres.

**Table 1.** Microscale combustion calorimetry and CIE colour space values (means and standard deviations) obtained from fibres of two white and two brown cottons

Cotton line	Colour	Microscale combustion calorimetry					CIE colour space		
		Char yield (%)	HRC (J g^−1^ K^−1^)	pHRR (W g^−1^)	tHR (kJ g^−1^)	Temp (°C) at pHRR	L*	a*	b*
MC-BL	Brown	24.6±0.2	112±2	144.0±1.4	6.1±0.1	359.1±1.4	49.39±0.48	12.50±0.30	29.96±0.47
MC-WL	White	16.6±0.8	150±7	195.7±9.2	8.2±0.3	362.0±1.1	89.40±0.20	-0.62±0.12	4.44±0.23
PD-3	White	18.1±0.5	134±4	175.9±6.4	7.8±0.1	356.1±1.5	91.93±0.90	-0.60±0.04	7.19±0.53
PD 93002	Brown	23.2±0.4	108±2	140.5±2.5	6.6±0.1	359.9±1.7	52.50±0.50	12.39±0.05	29.64±0.15

HRC: heat release capacity; pHRR: peak heat release rate; tHR: total heat release. L* indicates whiteness (0=black; 100=white); a* indicates colours from greenish (negative values) to reddish (positive values); and b* indicates colours from bluish (negative values) to yellowish (positive values).

**Fig. 1.**
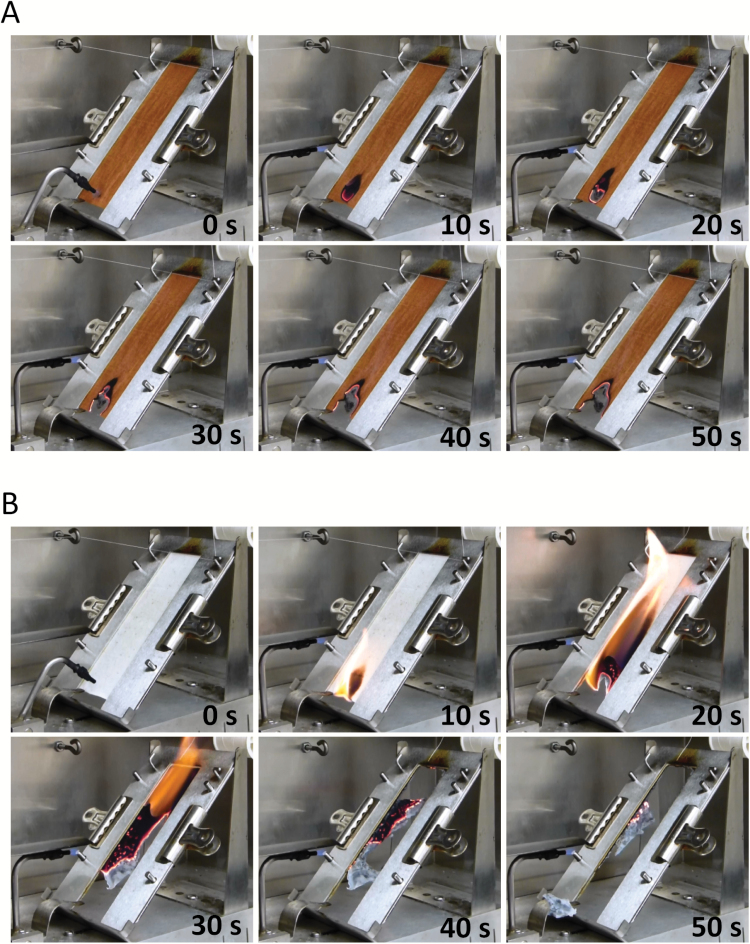
Time lapse depiction in 10s increments of the ASTM D1230-10 (45-degree) flammability test with nonwoven fabrics produced by hydroentanglement of cotton fibres harvested from *Lc1* and white cotton lines. (A) Following a 10s exposure to the ignition source, nonwoven fabrics produced from MC-BL fibres immediately self-extinguished with afterglow visible in the remaining panels. (B) Nonwoven fabrics produced from MC-WL fibres ignited and stopped the test timer at ~20s followed by complete consumption of the material by ~50s.

Nonwoven hydroentangled fabrics manufactured from fibres of white MC-WL and brown MC-BL were subjected to ASTM D1230-10 (ASTM, 2010) standardized testing for measuring the flammability of apparel textiles (Fig. 1). Fabrics manufactured from fibres of MC-BL self-extinguished immediately following removal of the flame source in all five replications (Fig. 1A). Afterglow, which is the continuation of glowing parts of a specimen after flame exposure has ceased, continued for 577.4±52.7s until the MC-BL fabrics were completely consumed and only char remained. Nonwoven hydroentangled fabrics manufactured from fibres of MC-WL ignited and the fabric was completely consumed with the time to break the thread recorded at 20.1±2.1s (Fig. 1B).

### Flame retardancy (FR) is independent of brown colour

**Fig. 2.**
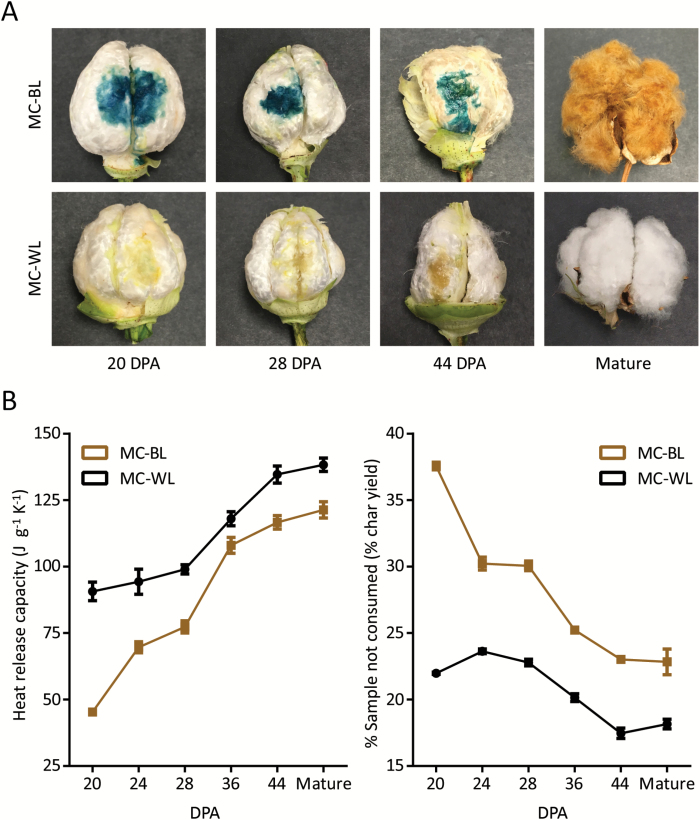
Accumulation of flavonoid PA precursor molecules demonstrated by DMACA staining is correlated with enhanced flame retardancy (lower heat release capacity) and elevated levels of inorganic material after pyrolysis (% char yield). (A) Bolls from the cotton lines MC-BL and MC-WL with the carpels removed and fibres stained with DMACA and mature fibres. (B) Comparative analysis of fibre flammability (heat release capacity) and inorganic material remaining after pyrolysis (% char yield) from developing and mature fibre of the cotton lines MC-BL and MC-WL. Developmental stages are indicated by days post-anthesis (DPA) on the x-axis. Error bars represent standard deviation of three biological replicates.

Developing *Lc1* fibres from the cotton lines MC-BL and PD 93002 revealed no brown colour development until approximately 36–44 DPA (Fig. 2). Immature cotton locules from a developmental time course were stained using DMACA, which revealed the presence of flavonoids in developing, and still colourless, *Lc1* fibres (Fig. 2A; Supplementary Fig. S1A). Unstained fibres harvested from the same bolls were analysed using MCC, which revealed the enhanced FR of *Lc1* fibres was already present while the fibres were colourless (Fig. 2B and Supplementary Fig. S1B). During development, the fibres of MC-BL and PD 93002 maintained consistently greater FR compared with their white counterparts (Fig. 2B and Supplementary Fig. S1B). Gravimetric data obtained from the MCC analysis also revealed a consistently higher percentage of unconsumed sample following pyrolysis (% char yield) (Fig. 2B and Supplementary Fig. S1B).

### Flame retardancy (FR) and brown colour are linked to 1.4Mb inversion

**Fig. 3.**
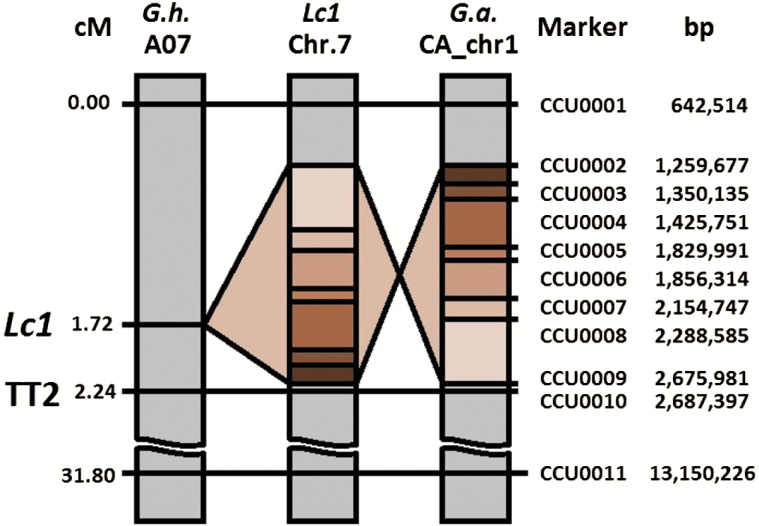
Linkage and physical maps of the *Lc1* genetic locus in *Gossypium hirsutum*. Genetic distances on *G. hirsutum* chromosome A07 are shown in centimorgans (cM) and physical distance along the orthologous *G. arboreum* reference sequence is shown in base pairs (bp). See also Supplementary Fig. S2 for sequencing-based evidence for the 1.4Mb genomic inversion.

**Fig. 4.**
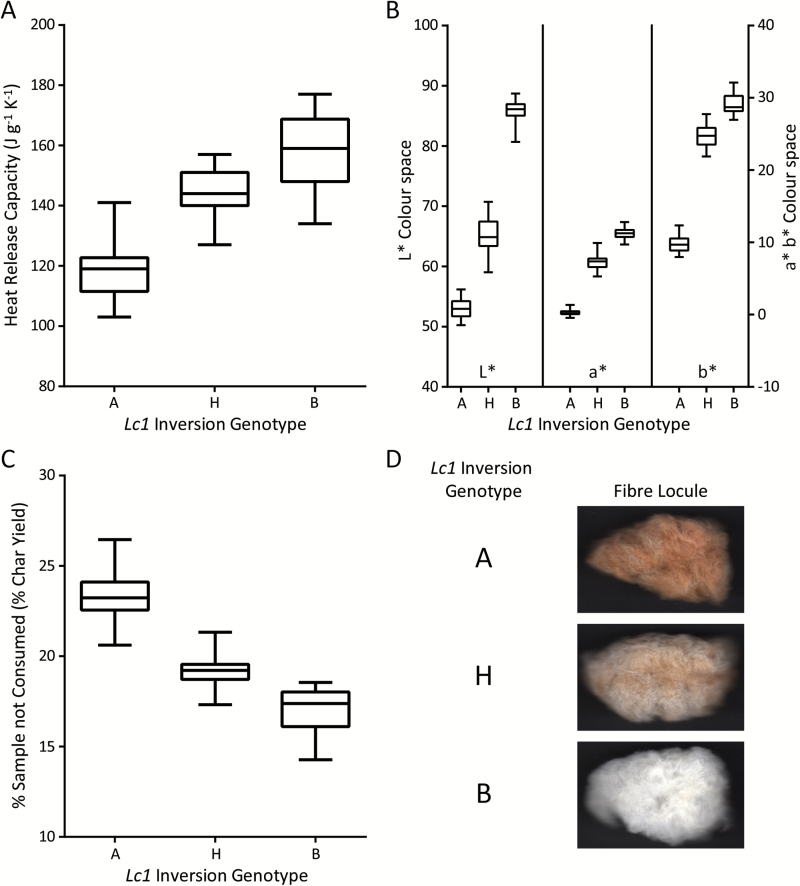
Correlations between CIE L*a*b* colour space and fibre flammability (heat release capacity) in a subset of the F_2_ segregating population. A lower HRC indicated a more flame retardant material. Whisker bars represent the maximum and minimum of three measurements. (A) Heat release capacity increased in order from homozygous *Lc1* fibres (A genotype) to heterozygous *Lc1* fibres (H phenotype) to homozygous wild-type (B genotype). (B) The CIE L*a*b* colour space coordinated with increased HRC starting with dark brown fibres (A genotype) to light brown fibres (H genotype) to white wild-type fibres (B genotype). (C) Percentage of sample not consumed (% char yield), which indicates the level of inorganic material remaining after pyrolysis, decreased in order from homozygous *Lc1* fibres (A genotype) to heterozygous *Lc1* fibres (H phenotype) to homozygous wild-type (B genotype). (D) Scanned images of single locules representative of the indicated *Lc1* inversion genotype. In CIE L*a*b* colour space, L* indicates whiteness (0=black; 100=white); a* indicates colours from greenish (negative values) to reddish (positive values); and b* indicates colours from bluish (negative values) to yellowish (positive values).

Whole-genome sequencing identified SNPs between the brown and white parental lines. The genomic sequencing data were submitted to the National Center for Biotechnology Information–Sequence Read Archive (NCBI-SRA) under the BioProject accession PRJNA326737. We designed SNP markers that we screened for polymorphism on a subset of the F_2_ populations. One marker, in *GhTT2_A07* (CCU0011), showed incomplete linkage with the brown phenotype. Manual inspection of the alignment files revealed the presence of a large 1.4Mb inversion, just upstream of *GhTT2_A07*. The inversion was present in the A-subgenomes of both *Lc1* cotton lines MC-BL and PD 93002, but was present in the wild-type orientation in the genomes of the white fibre cotton lines MC-WL and PD-3 (Supplementary Fig. S2). We designed and tested markers for the inversion junctions (CCU0002 and CCU0009) along with additional SNPs. We found that the inversion and the SNPs within the inversion exhibited complete genetic linkage to the brown phenotype in all 595 F_2_ plants (Fig. 3). The brown parental allele was homozygous in all 166 dark brown plants, heterozygous in all 273 light brown plants, and absent in all 157 white F_2_ progeny (Fig. 4B). Heterozygocity for the inversion also resulted in an intermediate flame retardancy as measured by MCC, as well as an intermediate level of inorganic material as measured by percentage char yield in the segregating progeny (Fig. 4).

### Activation of *GhTT2_A07* results in activation of flavonoid metabolic pathway

**Fig. 5.**
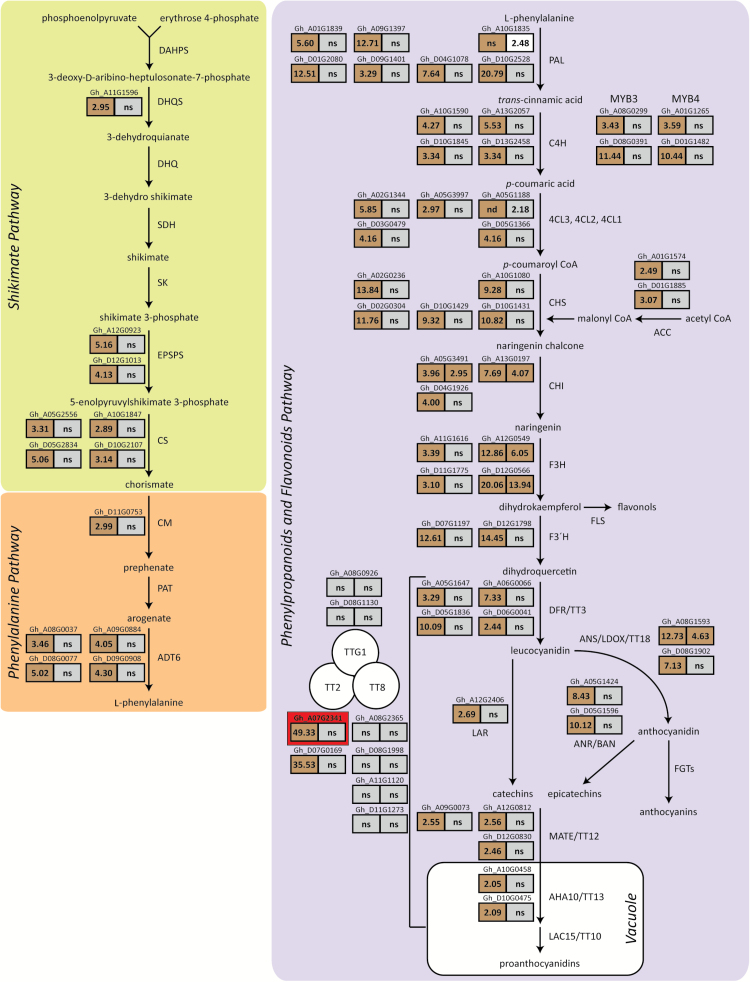
Differential expression of structural and regulatory genes in the committed phenylpropanoid and flavonoid pathways, and in the upstream phenylalanine and shikimate pathways. Genes that were statistically significant and upregulated more than 2-fold based on the mRNA-seq analysis are indicated along with the accession numbers based on the *Gossypium hirsutum* cv. TM-1 draft genome. The fold differences in expression between MC-BL and MC-WL are indicated in the boxes below the accession numbers, with 8 days post anthesis (DPA) in the left box and 20 DPA in the right. The boxes are colour-coded brown for upregulation in MC-BL and white for upregulation in MC-WL. No statistically significant difference in gene expression is indicated by ns. The genes known to be directly regulated by the TT2-TT8-TTG1 regulon in Arabidopsis are indicated within the bracket next to the regulatory complex in the illustration. The *GhTT2_A07* gene (Gh_A07G2341) activated by the upstream genomic inversion is indicated within the red box.

Transcriptome analysis by mRNA-seq revealed many of the genes in the phenylpropanoids pathway, beginning with the first committed step of phenylalanine ammonia-lyase (PAL), were significantly upregulated in developing fibres of MC-BL compared with MC-WL (Fig. 5). Genes in the shikimate and phenylalanine metabolic pathways upstream of the phenylpropanoids pathway were similarly upregulated at 8 DPA in developing fibres of MC-BL compared with MC-WL (Fig. 5). Twelve regulatory and structural genes in the flavonoids biosynthetic pathway were selected for verification of the mRNA-seq data and analysed by RT-qPCR that included additional developmental time points from all four cotton lines used in the study. We observed coordinated upregulation of many of the analysed genes in *Lc1* fibres, which peaked during fibre elongation and declined after 14 DPA (Supplementary Figs S3 and S4). An annotated list of all genes that exhibited statistically significant and differential expression (≥2-fold) at 8 and/or 20 DPA in fibres of the cotton lines MC-BL and MC-WL based on the mRNA-seq analysis are shown in Supplementary Table S4. The mRNA-seq data was submitted to the NCBI-SRA under the BioProject accession PRJNA326737.

### Altered metabolome in *Lc1* fibres

Gravimetric data obtained from the MCC analysis revealed a higher percentage of unconsumed sample following pyrolysis (% char yield). Increased levels of inorganic elements were previously observed in mature brown cotton fibres compared with white fibres and were observed to correlate with enhanced FR presumably through the formation of flavonoid–metal complexes (Hinchliffe *et al.*, 2015; Nam *et al.*, 2016). In the present study, iron, potassium, magnesium, and sodium were significantly higher in fibres of both *Lc1* cotton lines compared with the fibres of the white lines, with potassium being the most concentrated in the fibres among the elements analysed (Table 2).

**Table 2.** Element concentrations (means and standard deviations) in parts per million (ppm) in mature fibre of Lc1 cotton lines MC-BL and PD 93002 compared with wild-type lines MC-WL and PD-3

Element	MC-BL	MC-WL	PD 93002	PD-3
B	14.8±5.1	6.8±2	18.7±7.2	11.4±6.3
Ca	1503.3±192.5	1233.6±228	2993.8±1207.8	1211.1±361.6
Cu	2.1±1.7	0.7±0.1	1±0.1	0.5±0.2
Fe*	6.2±0.9	4.2±1.1	5.5±0.7	4.3±1.6
K*	14249.9±3319.4	8859.7±1815.4	16738.9±3470.7	7342±1595
Mg*	1358.8±196	1022.6±155.1	2031.5±580.6	1137.5±202
Mn	4.1±0.4	1.8±0.2	3.3±0.4	2.6±0.7
Na*	51.4±4.6	39±5.1	59.4±18.1	32.1±3.9
P	211.5±23.1	175.2±31	201.7±28.9	164.4±31.5
Si	16.3±11.6	10.3±3.4	12.3±2.8	7.8±0.8
Zn	2.6±0.6	1.6±0.2	2.3±0.8	1.6±0.4

*Significantly higher in *Lc1* lines compared with wild-type at the 0.05 probability level as determined by 2-tailed, paired *t*-test.

Data are representative of fibres collected in triplicate from two growing seasons. B: boron; Ca: calcium; Cu: copper; Fe: iron; K: potassium; Mg: magnesium; Mn: manganese; Na: sodium; P: phosphorous; Si: silicon; Zn: zinc.

## Discussion

The primary objective of this study was to identify the causative mutation resulting in brown colour and enhanced FR in the fibres and fabrics produced from *Lc1* cotton plants. The more long term objectives of this project are to try and develop a white fibre cotton plant with enhanced FR properties for use in specific textile applications that require a certain level of FR as dictated by regulatory authorities. This would include, but is not limited to, apparel, bedding, and automotive and aircraft interiors. This scope of application is dictated by standardized testing procedures and could include direct usage or minimization of additive FR compounds to a textile substrate. Identification of the gene responsible for the *Lc1* phenotypes was the logical first step in this process in order to better understand the regulation of the PA pathway and access the feasibility of separating brown colour from the FR phenotype of *Lc1* cotton fibres.

### A colourless flavonoid is the likely cause of natural flame retardancy in *Lc1* brown cotton

The accumulation of both flavonoid precursors and polymerized flavonoids that form PAs may contribute to enhanced FR in *Lc1* brown cotton fibres by coordination of metals with the B-ring of the flavonoid structure and the formation of flavonoid–metal complexes (Nam *et al.*, 2016). In this study the enhanced FR of fibres and nonwovens textiles of two white fibre and two brown fibre *Lc1* cotton lines was examined by MCC and confirmed by standardized textile flammability testing (Table 1 and Fig. 1). The enhanced FR of *Lc1* fibres was further demonstrated to exhibit consistently enhanced FR during development and prior to polymerization of flavonoid precursors into PAs (Fig. 2 and Supplementary Fig. S1). This novel observation of an immature white cotton fibre exhibiting enhanced FR independent of the brown fibre colour implicates a colourless compound in the mechanism of enhanced FR. While flavonoid–metal complexes that include brown coloured PAs may contribute to the enhanced FR in mature brown fibres, the existence of enhanced FR in the colourless immature fibres suggests that one or more colourless flavonoids or PA precursors can impart natural FR to developing cotton fibres.

### The causative *Lc1* mutation was mapped to a non-coding genomic inversion

Here we report the complete linkage of a 1.4Mb genomic inversion with the brown colour and the enhanced FR phenotypes in *Lc1* fibres. This is the first report that identified the causative mutation of a naturally coloured cotton line, and also provided the first insights into the regulatory mechanism of the PA biosynthetic pathway in cotton fibres. Upregulation of the *GhTT2_A07* transcription factor gene just downstream of the inversion suggests this non-coding region of the genome may contain regulatory elements that either suppress *GhTT2_A07* expression in the wild-type orientation, or activate its expression in the mutant *Lc1* orientation.

### Upregulation of metabolic pathways by activation of *GhTT2_A07* in *Lc1* fibres

In the current study, as a result of the activation of *GhTT2_A07*, additional upstream effects were observed in the PA pathway that included upregulation of all genes in the phenylpropanoid and flavonoid pathways, as well as genes in the shikimate pathways and phenylalanine pathways that provide the initial precursor compounds (Fig. 5). A discussion of the regulatory mechanisms of these pathways is beyond the scope of this research and extensively reviewed in Maeda and Dudareva (2012). The TT2 transcription factor is part of the TT2–TT8–TTG1 regulon that has been extensively studied in Arabidopsis and directly regulates late biosynthetic pathway genes in the PA pathway (Fig. 5 and Supplementary Table S5) (Xu *et al.*, 2014*a*,b). Orthologues of TT8 and TTG1 were expressed at much lower levels than *GhTT2_A07* and their transcript abundances were not significantly different in *Lc1* fibres compared with white fibres in the mRNA-seq and RT-qPCR analyses (Fig. 5 and Supplementary Figs S3 and S4). The absence of coordinated expression of the genes comprising this regulatory complex suggests that *GhTT2_A07* may act independently or in conjugation with unknown transcription factors in upregulating the PA pathway in *Lc1* fibres or that the accumulation of downstream metabolites initiates positive feedback regulation. The activation of *GhTT2_A07* by the genomic inversion in *Lc1* cotton results in the expression of many genes that may play critical roles in the development of natural flame retardance.

### Future work will target downstream genes to restore colourless fibres but maintain FR

Currently, we are targeting late biosynthetic genes in the committed PA pathway using a virus-induced gene silencing (VIGS) system that was previously demonstrated to be usable as a functional assay in cotton fibres (Wan *et al.*, 2016). Based on our results that demonstrated enhanced FR in developing colourless *Lc1* fibres, inhibition of specific late biosynthetic genes involved in PA precursor biosynthesis, transport, and/or polymerization to PAs may allow accumulation of flavonoid precursors and flavonoid–metal complexes and result in the enhanced FR phenotype independent of brown colour.

## Supplementary data

Supplementary data are available at *JXB* online.

Figure S1. Enhanced flame retardancy develops prior to brown colour in *Lc1* fibres

Figure S2. Alignment of DNA reads to pseudo-tetraploid reference genome reveals the 1.4Mb genomic inversion upstream of *GhTT2_A07* in *Lc1* cotton lines.

Figure S3. A RT-qPCR comparative analysis of the transcript abundances of selected genes in the phenylpropanoid and ﬂavonoid pathways from developing ﬁbres of the cotton lines MC-BL and MC-WL.

Figure S4. A RT-qPCR comparative analysis of the transcript abundances of selected genes in the phenylpropanoid and ﬂavonoid pathways from developing ﬁbres of the cotton lines PD 93002 and PD-3.

Table S1. Inversion-speciﬁc marker scheme and ampliﬁcation results.

Table S2. SNP genetic markers used to map the 1.4Mb genomic inversion in *Lc1* cotton lines.

Table S3. Nucleotide sequences of primer pairs utilized for RT-qPCR, gene annotations, and database accession numbers.

Table S4. Significantly and differentially expressed genes from mRNA-seq analysis.

Table S5. List of enzyme abbreviations used in Fig. 5 and Supplementary Figs S3 and S4.

Supplementary Data
