# Supplementary material for: The GhTT2_A07 gene is linked to the brown colour and natural flame retardancy phenotypes of Lc1 cotton (Gossypium hirsutum L.) fibres
Source: J Exp Bot. 2016 Aug 27;67(18):5461–71. doi: 10.1093/jxb/erw312 (PMC5049394; doi:10.1093/jxb/erw312)
Supplement: Supplementary Data [file supp_67_18_5461__index.html]

The GhTT2\_A07 gene is linked to the brown colour and natural flame retardancy phenotypes of Lc1 cotton (Gossypium hirsutum L.) fibres — The GhTT2\_A07 gene is linked to the brown colour and natural flame retardancy phenotypes of Lc1 cotton (Gossypium hirsutum L.) fibres — Supplementary Data 

# The *GhTT2\_A07* gene is linked to the brown colour and natural flame retardancy phenotypes of *Lc1* cotton (*Gossypium hirsutum* L.) fibres

## Supplementary Data

Data files

- Supplementary\_Figures\_S1\_S4.pdf - Supplementary Data
- Supplementary\_Tables\_S1\_S3\_S5.pdf - Supplementary Data
- Supplementary\_Table\_S4.xlsx - Supplementary Data
