# Supplementary material for: The GhTT2_A07 gene is linked to the brown colour and natural flame retardancy phenotypes of Lc1 cotton (Gossypium hirsutum L.) fibres
Source: J Exp Bot. 2016 Aug 27;67(18):5461–71. doi: 10.1093/jxb/erw312 (PMC5049394; doi:10.1093/jxb/erw312)
Supplement: Supplementary Data [file supp_erw312_Supplementary_Figures_S1_S4.pdf]

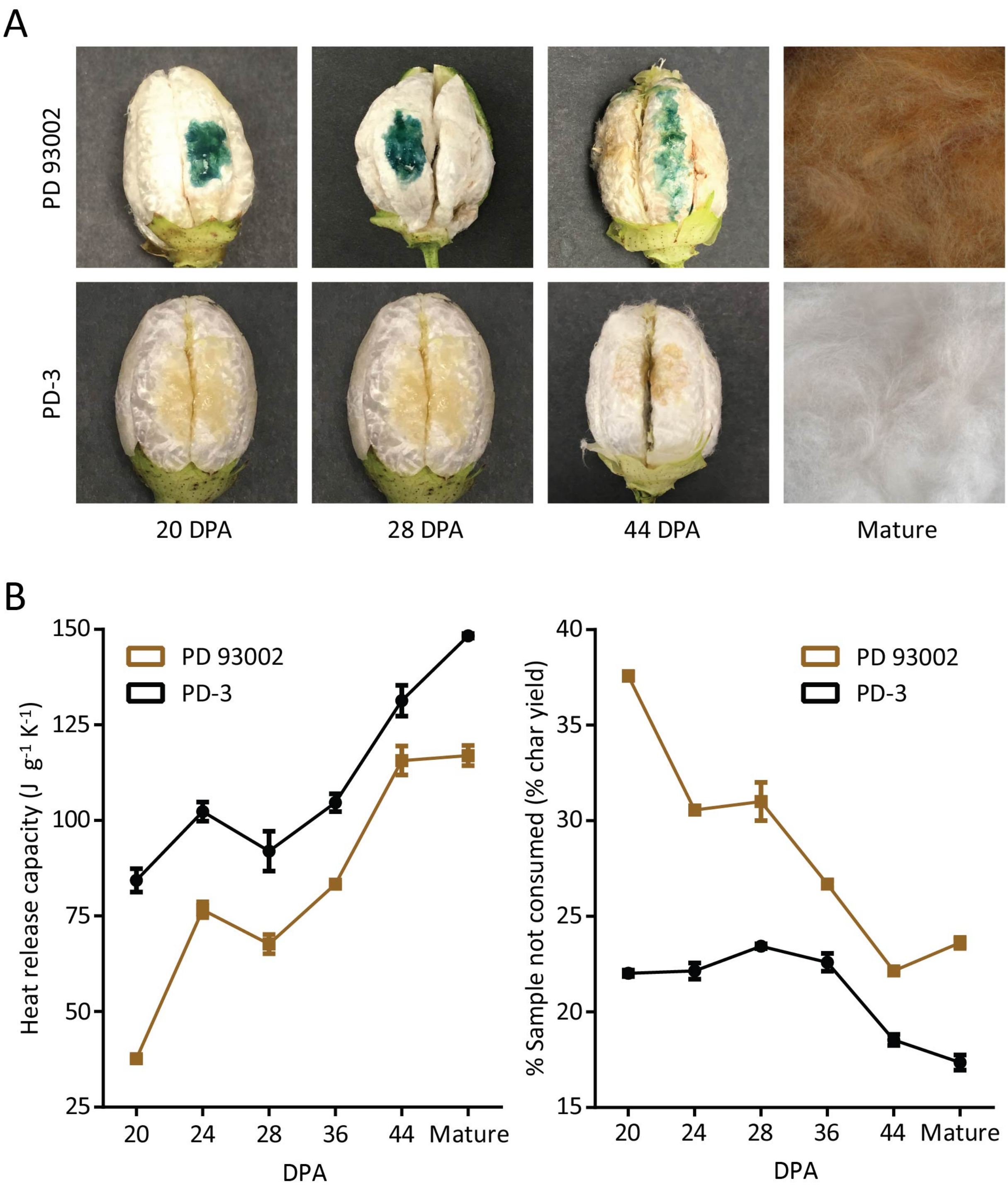

Fig. S1. Enhanced flame retardancy develops prior to brown colour in *Lc1* fibres. Accumulation of flavonoid PA precursor molecules demonstrated by DMACA staining is correlated with enhanced flame retardancy (lower heat release capacity) and elevated levels of inorganic material after pyrolysis (% char yield). **(A)** Bolls from the cotton lines PD 93002 and PD-3 with the carpels removed and fibres stained with DMACA and mature fibres. **(B)** Comparative analysis of fibre flammability (heat release capacity) and inorganic material remaining after pyrolysis (% char yield) from developing and mature fibre of the cotton lines PD 93002 and PD-3. Error bars represent standard deviation of three biological replicates.

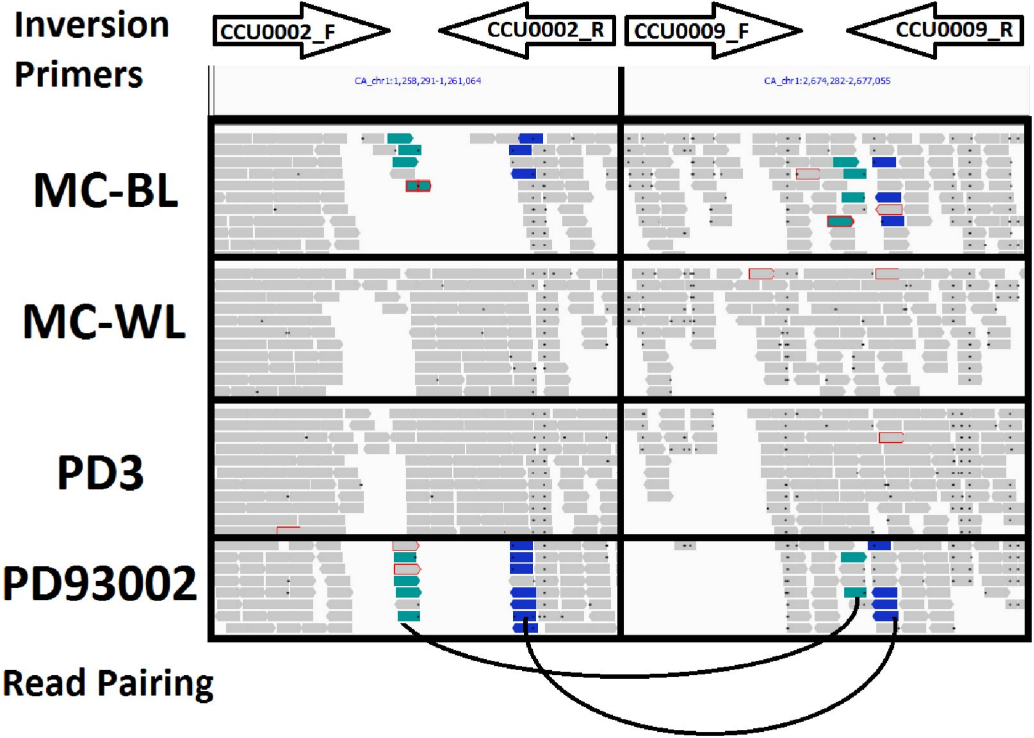

Fig. S2. Alignment of DNA reads to pseudo-tetraploid reference genome reveals the 1.4 Mb genomic inversion upstream of *GhTT2_A07* in *Lc1* cotton lines.

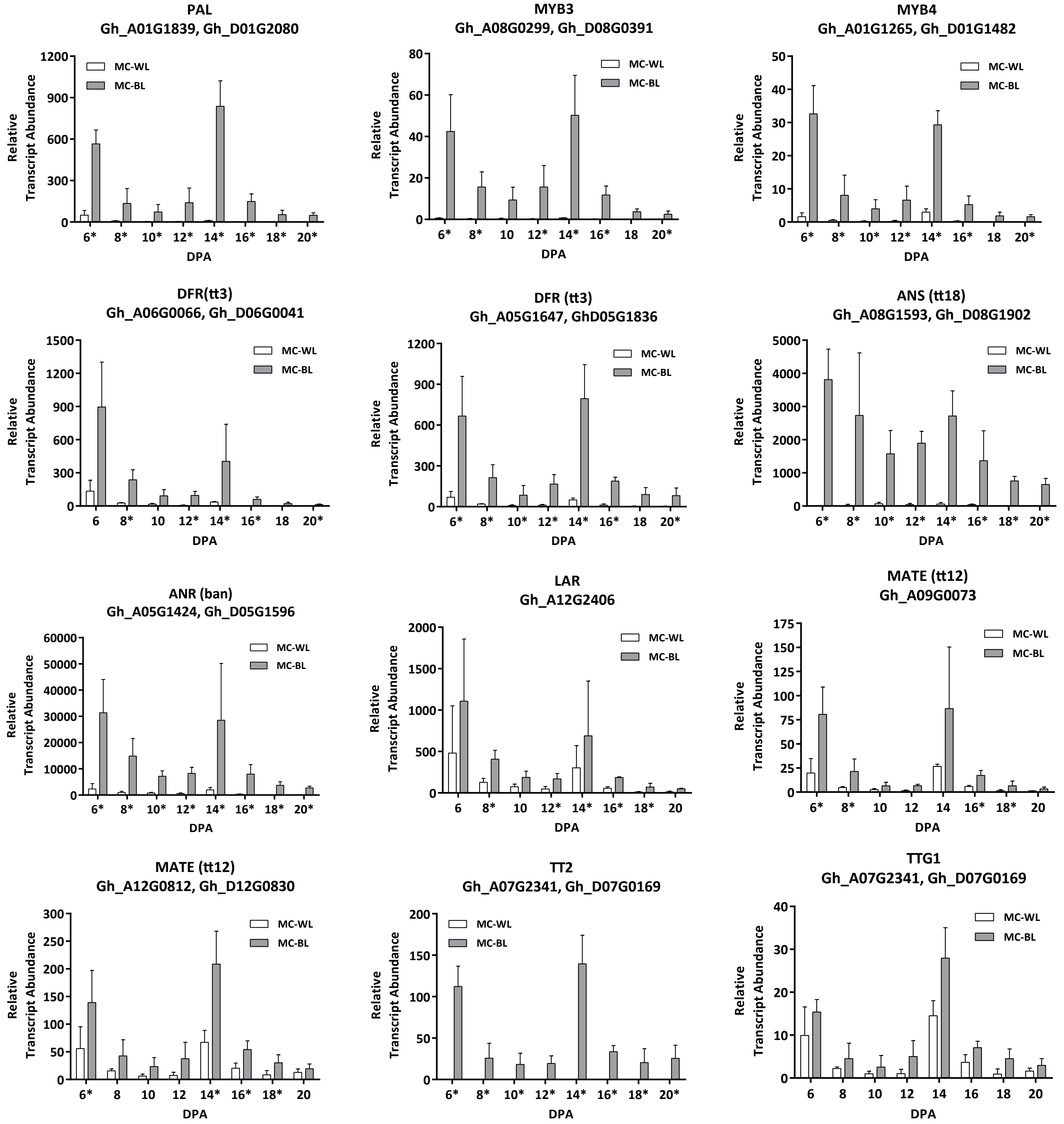

Fig. S3. A RT-qPCR comparative analysis of the transcript abundances of selected genes in the phenylpropanoid and flavonoid pathways from developing fibres of the cotton lines MC-BL and MC-WL. The relative transcript abundances of all genes, timepoints and cotton lines were normalized to TTG1 from 12 DPA MC-WL fibres. * indicates greater than 2-fold difference in transcript abundance that is statistically significant at the 0.05 probability level as determined by paired t-test. The gene abbreviations can be found in Table S5.

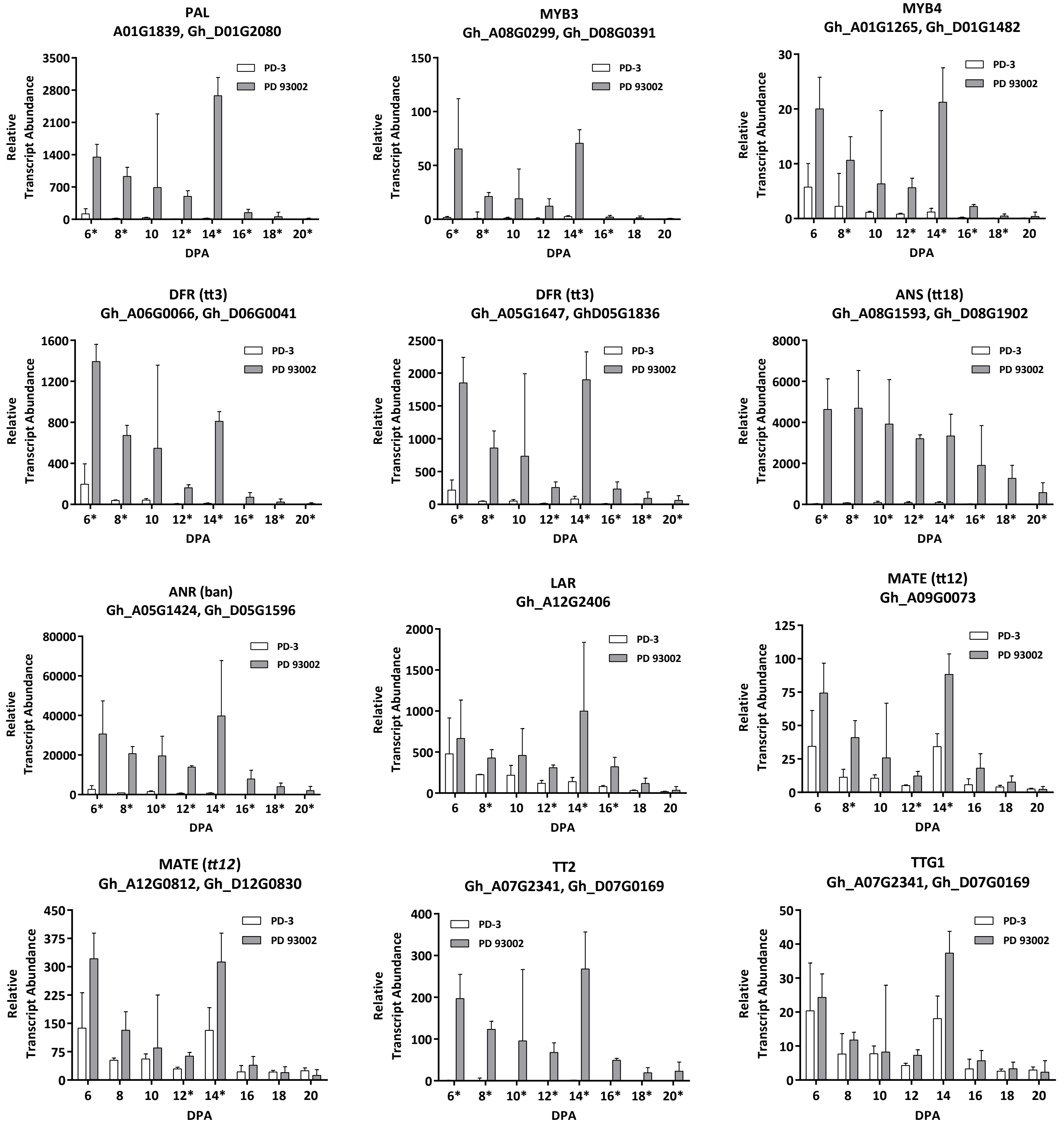

Fig. S4. A RT-qPCR comparative analysis of the transcript abundances of selected genes in the phenylpropanoid and flavonoid pathways from developing fibres of the cotton lines PD 93002 and PD-3. The relative transcript abundances of all genes, timepoints and cotton lines were normalized to TTG1 from 12 DPA MC-WL fibres. * indicates greater than 2-fold difference in transcript abundance that is statistically significant at the 0.05 probability level as determined by paired t-test. The gene abbreviations can be found in Table S5.
