# Supplementary material for: The GhTT2_A07 gene is linked to the brown colour and natural flame retardancy phenotypes of Lc1 cotton (Gossypium hirsutum L.) fibres
Source: J Exp Bot. 2016 Aug 27;67(18):5461–71. doi: 10.1093/jxb/erw312 (PMC5049394; doi:10.1093/jxb/erw312)
Supplement: Supplementary Data [file supp_erw312_Supplementary_Tables_S1_S3_S5.pdf]

Table S1. Inversion-specific marker scheme and amplification results. Primer sequences are provided in Table S2.

	SNP Primer Combinations			
	CCU0002_F	CCU0009_F	CCU0002_F	CCU0002_R
Cotton Lines	CCU0002_R	CCU0009_R	CCU0009_F	CCU0009_R
MC-BL	-	-	+	+
MC-WL	+	+	-	-
PD-3	+	+	-	-
PD930002	-	-	+	+
Heterozygote	+	+	+	+

Table S2. SNP genetic markers used to map the 1.4 Mb genomic inversion in *Lc1* cotton lines.

	Primer Sequence		
Primer	Forward		Reverse
CCU0001	TGAAAGGCGGTTCTTTAGT		ATGTCCGAGATTGATTTAGATATG
CCU0002	CATTGGAGAATAAAGTCTCATGCT		TTCCAACATTATTTACCGGAATGG
CCU0003	ATTGGTCGCTCTTCCTGAT		ATATCCTCTGTAGAGGTATCT
CCU0004	TGCCGGTGACTTTCATG		CATGCATTTGTGTCATGCTCT
CCU0005	AAGCACTTTCCTGGATAGCA		GAAGATGAACCTATTCTTGACAG
CCU0006	CGTAAACTTTTACAATCCGAATTGTT		AAACTTGTTTGGCTTTAAAAGAGG
CCU0007	AGGGACAACATGTGGATAATAG		CTGTCTTTATTCTTGAGACAGATAGCAA
CCU0008	AATCATTCTCGAGCTCAATCA		GAAGAAAGAGAACGTACCTTTTGA
CCU0009	CATCCATACTAAGATCAATGGCG		CATATCAGCAAGAGCACACC
CCU0010w*	CTTGACACTGATGAATAGC		AGTAATTTTCTCATAACATGTAA
CCU0010b*	CTTGACACTGATGAATAGA		AGTAATTTTCTCATAACATGTAA
CCU0011	CTTGAATATCAAATGGGTTTGCA		CTGAATTTTCTTTGTTTTGTCATT

* w and b indicates primer pair specific for white wild-type fibres and *Lc1* fibres, respectively.

Table S3. Nucleotide sequences of primer pairs utilized for RT-qPCR, gene annotations, and database accession numbers. Gene product abreviations can be found in Table S5.

	Primer Sequence (5´ - 3´)		Gene Accessions	
Gene	Forward	Reverse	TAIR	TM-1*
PAL	TGAAATCGCAATGGCCTCCT	TGCTTCGGCTGTTTTTCGTG	AT2G37040.1	Gh_A01G1839; Gh_D01G2080
MYB3	AACAAGCCACGAACACGAGA	CGAAGGGTTTCCTCCTGCTT	AT1G22640.1	Gh_A08G0299; Gh_D08G0391
MYB4	ATGAGGAAGCCTTGTTGCGA	GCAACCAGCTTCACCGTTTT	AT4G38620.1	Gh_a01G1265; Gh_D01G1482
DFR (TT3)	CCACCATTGTTGAGCTTGCC	TCTCCACAGCTCCGACAAAC	AT5G42800.1	Gh_A06G0066; Gh_D06G0041
DFR (TT3)	GACCCTGCAGACAACTCGAA	GGACTCGAAGTCCATAGGCG	AT5G42800.1	Gh_A05G1647; Gh_D05G1836
ANS (TT18)	GTCGAAGCTCACACCGATATAA	ACCCACTTGCCTTGGTAAA	AT4G22880.1	Gh_A08G1593; Gh_D08G1902
ANR (BAN)	TCCAAGACGTTGGCTGAAA	GAAGGACCAGTCATGAGAGAAG	AT1G61720.1	Gh_A05G1424; Gh_D05G1596
LAR	GAGCAAGATTAGGAGGCAGATAG	TGGATGAGTGTTGTCATGGTAG	AT1G75290.1	Gh_A12G2406; Gh_D12G2642
MATE (TT12)	TTATGTTGGGCATGGCGAGT	CTTGAAGGACGGTGTCCGAA	AT3G59030.1	Gh_A09G0073; Gh_Sca011468G01
MATE (TT12)	AGTAGCGGGAATTTGGTGGG	AAGAGGCGACAGATGTTGGG	AT3G59030.1	Gh_A12G0812; Gh_D12G0830
TT2	ATTGTACAGTGATGGCGGCT	ATTGCCTCACCCAACGGAAA	AT5G35550.1	Gh_A07G2341; Gh_D07G0169
TTG1	ACGCTAACAGAGTCCACATAATC	AAGTTTGGTGGGTGGGTAAG	AT5G24520.1	Gh_A08G0926; Gh_D08G1130
GhUCP E2	CGGAAAGAGGTGAAGATGTCAAC	GGATCTTGCTGCAACCTCTTAAA	AT2G02760.1	Gh_A12G1527; Gh_D12G1650
GhTubA4	GATCTCGCTGCCCTGGAA	ACCAGACTCAGCGCCAACTT	AT1G50010.1	Gh_A05G3784; Gh_D05G2068
18S	CGTCCCTGCCCTTTGTACA	AACACTTCACCGGACCATTCA	AT3G41768.1	na

* The *G. hirsutum* TM-1 subgenome gene accessions are from the assembly of Zhang *et al.*, 2015. Full sequence information is available at www.cottongen.org.

Table S5. List of enzyme abbreviations used in Fig. 5 and Supplementary Figs S3 and S4.

	Enzyme Abbreviation	Enzyme Name
Shikimate Pathway	DAHPS	3-Deoxy-wrabineheptulosonate 7-phosphate synthase
	DHQS	3-Dehydroquinate synthase
	DHQ	3-Dehydroquinate dehydratase/shikimate dehydrogenase
	SDH	shikimate dehydrogenase
	SK	Shikimate kinase
	EPSPS	5-Enolpyruvylshikimate 3-phosphate (EPSP) synthase
	CS	Chorismate synthase
Phenyalanine Pathway	CM	Chorismate mutase
	PAT	Prephenate aminotransferase
	ADT	Arogenate dehydratase
Phenylpropanoid and flavonoid pathways	PAL	Phenylalanine ammonia lyase
	C4H	Cinnamate 4-hydroxylase
	4CL3, 2, 1	4-Coumarate-CoA ligase 3, 2, and 1
	CHS	Chalcone synthase
	ACC	Acetyl-CoA carboxylase
	CHI	Chalcone isomerase
	F3H	Flavonol 3-hydroxylase
	FLS	Flavonol synthase
	F3'H	Flavonoid 3'-hydroxylase
	DFR	Dihydrokaempferol 4-reductase
	ANS/LDOX/TT18	Anthocyanidin synthase/Leucoanthocyanidin dioxygensae/TRANSPARENT TESTA 18
	ANR/BAN	Anthocyanidin reductase/BANYULS
	LAR	Leucoanthocyanidin reductase
	FGT	Flavonol 3-O-glucosyltransferase
	MATE/TT12	Multi-drug and toxic efflux transporter/TRANSPARENT TESTA 12
	AHA10/TT13	AUTOINHIBITED H(+)-ATPASE ISOFORM 10/TRANSPARENT TESTA 13
	LAC15/TT10	Laccase 15/TRANSPARENT TESTA 10
	TT2 and TT8	TRANSPARENT TESTA 2 and 8
	TTG1	TRANSPARENT TESTA GLABRA 1
